# Internet Gaming Disorder in Early Adolescents: Gender and Depression Differences in a Latent Growth Model

**DOI:** 10.3390/healthcare9091188

**Published:** 2021-09-09

**Authors:** Rong Wang, Shuqi Yang, Yun Yan, Yu Tian, Peng Wang

**Affiliations:** School of Psychology, Shandong Normal University, Jinan 250358, China; Wangrong_0614@163.com (R.W.); yozukii@outlook.com (S.Y.); yanyun981019@outlook.com (Y.Y.); tianyurest@outlook.com (Y.T.)

**Keywords:** Internet gaming disorder, depression, gender, latent growth model

## Abstract

Background: Studies have shown that many Internet game players may have experienced Internet gaming disorder (IGD), which is thought to coexist with a variety of cognitive and psychological disorders, such as depression. A three-year, four-point longitudinal tracking study was conducted to examine the trajectory of IGD of Chinese early adolescents, as well as the predictive level of gender and depression for IGD. Participants (*N* = 316) completed questionnaires assessing IGD and depression at the time of the T1, T2, T3, and T4. This study adopted the widely used latent growth model for data analysis. The results showed that, in the early stage of adolescence, (1) the development trajectory of individual IGD was shown as a nonlinear latent variable growth model; the IGD was significantly higher than zero when teenagers were first measured (sixth grade); and, while on the rise, its growth rate is gradually slowing. (2) Gender can significantly predict the development trajectory of individual IGD. (3) Individuals with a high initial level of depression usually have a high initial level of IGD, individuals with a large range of depression display a large range of IGD, and those with a rapid rate of depression change show similar change in their IGD. In sum, this study provided an empirical basis for the prevention and intervention of IGD in early adolescents.

## 1. Introduction

With the development of the Internet, global network usage has increased from 1.99 billion in 2010 to 3.385 billion in 2016 [1]. According to the 47th China Statistical Report on Internet Development released by the China Internet Network Information Center [2], by December 2020, China had 0.989 billion Internet users. Adolescents aged 10–19 were among the major groups using the Internet, with the trend of network users shifting from adult groups to adolescent and older groups. As the use of the network among adolescent groups increases, studies have indicated that adolescents are experiencing the risk of Internet use disorders or Internet addiction [3]. Over the past two decades, many studies have focused on the clinical phenomenon of online use disorders, the most concerning of which is Internet gaming disorder (IGD) [4].

The American Psychiatric Association (APA) has identified IGD as a potential mental disorder [5]. At the same time, in the fifth edition of the *Diagnostic and Statistical Manual* of Mental Disorders (DSM-5), IGD is included as a specific network addiction disorder, emphasizing that this situation is highly likely to be clinically significant [4].

IGD and potential neurobiological mechanisms are similar to other network addictions, such as symptoms of IGD that manifest as specific responses to game-related cues that lead to decreased impulse and cognitive control [6]. IGD has a negative impact on individual physical health, mental health, well-being, daily behavior, and so on [7,8]. At the same time, individuals with IGD showed lower psychosocial elasticity, higher stress perception, higher levels of depression [9], and some other nonpsychotic psychological symptoms (anxiety, impulsivity) and physical aggression [10].

Studies have shown that many Internet game players may have experienced IGD [5], while epidemiological surveys have revealed that IGD affects up to 3% of adolescents, with a higher proportion in Asia (10.3% in China) [8], which is becoming an important issue among the youth community. As a kind of health problem, it is necessary to strengthen the research on the development and distribution of IGD among different groups of teenagers. Pontes et al. (2016) [11] indicate that the prevalence rate of IGD among 13 year olds in the United Kingdom is on average 2.5%, and 3.8% in German adolescents of the same age. IGD initially seems to develop in individuals in early adolescence [12]. At the same time, with the development of individual physiological age, the level of IGD also shows an increasing trend. For example, Wichstrøm et al. (2019) [13] found the prevalence of IGD to be 1.7% in Norwegian 10 year olds, and the prevalence estimate for IGD in German adolescents was 3.5% (Mage = 14.58) [14]. However, the relationship between the rate of change of individual IGD and physiological age in early teens has not been studied.

At the same time, cultural differences need to be taken into account in the development of prevention and intervention strategies for IGD [15]. Therefore, to explore the development process of IGD, this study focuses on the characteristics of IGD in the early stages of development of young Chinese people. This study hypothesizes that its initial level is significantly greater than 0, with a significant growth trend, and the quadratic change will gradually increase.

As an addictive behavior, IGD is thought to coexist with a variety of cognitive and psychologic disorders, such as depression and attention deficit hyperactivity disorder [16]. Furthermore, depression is included as a common psychological abnormality in the variables in this study. The link between depression levels and IGD has been proven by many studies. Choi et al. (2017) [17] noted that depression is prevalent among those with IGD, and that the severity is significantly associated with an increase in IGD [18]. However, the specific longitudinal link between depression and IGD remains unclear.

Männikkö et al. (2020) [19] reported positive effects of IGD behavior on depression, anxiety, obsessive compulsive disorder, and physicalization. People with a higher level of stress and depression are more likely to have IGD, because they probably cope with negative emotion through gaming [20]. At the same time, regression and moderation analyses revealed that depression was an individual risk factor for the development of online game disorders [21]. Based on the analysis of the aforementioned studies, there is a lack of longitudinal research on the relationship between IGD and depression, and it is not clear whether depression levels are the cause or consequence of IGD [22].

Brand et al. (2016) [4] proposed an Interaction of Person–Affect–Cognition–Execution (I-PACE) model, which consists of four variables that can cause disorder of network use, susceptibility variables (P), emotional and cognitive responses to internal or external stimuli (A, C), execution and suppression of controls, and decision behavior (E) that leads to the use of specific network applications/websites. Depression is incorporated as a psychological factor into the P component of the model (a susceptibility variable representing core personal characteristics) to predict specific network disorder behavior. The theoretical hypothesis of the I-PACE model still lacks the relevant practical research evidence, so this study explores the longitudinal correlation between depression level and IGD, regarding it as a dependent variable and depression as an independent one.

Numerous studies have found that adolescents show gender differences in behavior, psychological characteristics, and so on, such as higher levels of drug abuse in early teens, differences in perspective-taking ability, and empathy trends. Specifically, the trend increases more steeply for girls than for boys, and the level of empathy of girls is higher than that of boys [23]. In terms of early adolescence (12 to 15 years of age), there were found to be gender differences in depression levels and that they peak during this time and then decline and remain stable in adulthood [24]. At the same time, studies on the Internet have shown significant gender differences in the purpose and intensity of Internet use among junior high school students, with boys significantly more likely to use the Internet than girls [25]. Although there have been numerous studies demonstrating a significant correlation between IGD and gender (men have higher levels of IGD than women) [14,26,27], the specific predictive direction (starting level/trend/quadratic change) in the development of IGD is still unclear. Therefore, gender is included as an independent variable in the study model, and the association between gender and IGD is explored.

This study conducted a longitudinal survey of Chinese students (grade 6 through 9) over a period of 3 years and 4 points in time. Because longitudinal data have always played an important role in empirical research in psychology, there are many new and exciting analytical methods to investigate the differences in the development and change of inter- and intra-individuals. The research method chosen for this study is the broadly used latent growth model (LGM) [28], a term commonly used in the field of social sciences, which usually refers to a statistical method that allows for the estimation of inter-individual variability in the variability of intra-individual patterns [29]. Compared with other research methods, the LGM can optimally capture the collection of individual change trajectories over time.

In summary, the current study examines the change trajectory of IGD among young Chinese adolescents at the early stage through the LDMs. Furthermore, the study also examines the predictive levels of gender and depression for the trajectory of IGD.

This study formulates the following hypotheses:

**Hypotheses** **1** **(H1).** *The initial level of early IGD in adolescents is significantly higher than 0 and, over time, shows an increasing trend and accelerated growth.*

**Hypotheses** **2** **(H2).** *Gender can significantly predict the development trajectory of early IGD in adolescents.*

**Hypotheses** **3** **(H3).** *Early adolescent depression levels can significantly predict the development of IGD.*

## 2. Materials and Methods

### 2.1. Participants

The data are taken from longitudinal research on behavior problems relating to network and Internet use. The students were junior high school students from Shandong Province in East China, and represented four-year junior high schools (grade 6 to 9). The studies lasted three years and participants were tested four times. The first test was conducted in the first term before the mid-term examination. The teacher issued the “Guardian Informed Consent” to introduce the study, which was signed by the guardian and collected by the teacher and forwarded to the researchers. The next three tests were arranged before the second, third, and fourth exams, respectively. A total of 316 students (150 boys and 166 girls, with an initial average age of 11.63 ± 0.64) completed all four surveys.

Because the LGM allows for tracking of missing values of data and the ability to estimate missing values in the model [30], the sample included the participants with missing values for the dependent variable (IGD) at different times (IGD) at the time of the T1, T2, T3, and T4 tests.

### 2.2. Measures

#### 2.2.1. Internet Gaming Disorder

The IGD scale was translated and revised [31], and the Chinese version was obtained. The scale contains 10 items, such as “When you haven’t played a game for a long time, do you often think of the scenes you used to play or are you looking forward to another game?” Items were scored according to a seven-point scale, with 1 for “strongly disagree” and 7 for “very much agree.” The higher the total score, the higher the level of online gaming disorder. The Cronbach’s α coefficient of the scale was 0.920.

#### 2.2.2. Depression

The Chinese version of the Center for Epidemiological Studies Depression Scale (CES-D) prepared by Radloff (1977) was used to evaluate the frequency of current depressive symptoms, focusing on depressive emotions or moods. The scale consists of 20 items with a score of 0 to 3, and the higher the score, the more frequent the appearance of depressive symptoms. Local researchers in China have proved that this questionnaire has good structural validity in adolescents and can be used to measure the depression level of adolescents [32]. The Cronbach’s α coefficient of the scale was 0.840.

### 2.3. Data Analysis

SPSS 21.0 (Shandong Normal University, Jinan, China) and Mplus 7.0 (Shandong Normal University, Jinan, Chin) were used to perform descriptive statistics and build a growth model of potential variables to test the development trajectory of youth IGD. In the process of constructing the LGM, the unconditional model is first constructed, which forms the repeated measurement of the IGD at four points in time. Intercepts represent the baseline state of an individual, which is the level of observation measured in the first measurement. Slope is used to represent differences in trajectories between individuals.

This study examines the trajectory of the change of IGD, and whether there are significant individuals in the starting level, change trends, and quadratic change. On this basis, gender (a time-invariant covariate) and depression (a time-varying covariate) were added to the model, and the predictive function of different genders and different extent of depression on the change trajectory of the IGD in early adolescents was investigated.

### 2.4. Missing Data Analyses

Missing data were analyzed using the SPSS 21.0 EM estimation option, and the results indicate that χ^2^ = 363.856, *df* = 337, *p* = 0.151, which indicates that the missing data are missing completely at random (MCAR) [33]. This means the probability that a variable absence occurs is independent of the variable itself and other variables, and the event in which the variable is missing is a random event [34]. Following Muthén (2012) and Zhang and Chen (2017), this study adopts the multi-interpolation method favored by scholars [34,35]. Specifically, the Data COMMAND in Mplus 7.0 (DATA IMPUTATION) fills in the missing data MCAR. That is, in the case of completely random missing data, the missing data are interpolated with two or more values that reflect the probability distribution of the data themselves [35].

## 3. Results

### 3.1. Preliminary Analysis

Mean levels of main variables and bivariate correlations are presented in Table 1. There was a significant positive correlation between the level of depression and the level of IGD at each time point (*r* = 0.271, *p* < 0.001; *r* = 0.351, *p* < 0.001; *r* = 0.278, *p* < 0.001; *r* = 0.273, *p* = 0.001). Meanwhile, gender was significantly correlated with IGD (*r* = 0.129, *p* = 0.035).

### 3.2. Trajectories of Internet Gaming Disorder in Early Adolescence

The unconditional growth model was tested. Comparing linear and quadratic LGMs revealed the quadratic model fit the data significantly better than the linear model. The model fit of the linear LGM model was *χ^2^* = 82.12, *df* = 5, *p* < 0.001, *CFI* = 0.368, *TLI* = 0.242, *SRMR* = 0.240. The model fit of the quadratic LGM model was *χ^2^* = 1.007, *df* = 1, *p* = 0.3155, *CFI* = 1.000, *TLI* = 1.000, *SRMR* = 0.014.

**Figure 1:**
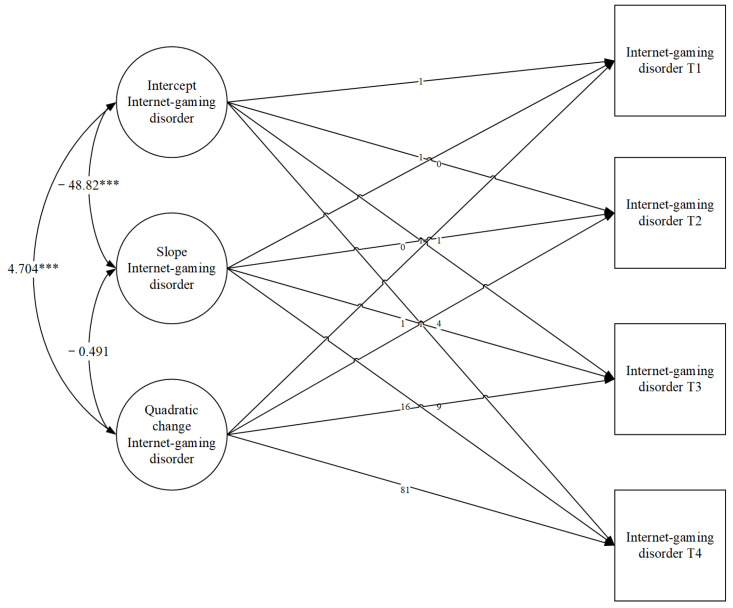
Unconditional quadratic LGMs. *** *p* < 0.001.

The quadratic growth model (see Figure 1) showed that the mean intercept of adolescents’ IGD in T1 (sixth grade) was significantly different from zero (*M* _intercept_ = 20.76, *SE* = 0.63, *p* < 0.001; *V*_intercept_ = 111.33, *SE* = 14.27, *p* < 0.001). Consistent with our prediction, IGD showed a statistically significant increase from sixth grade to ninth grade (*M* _slope_ = 1.50, *SE* = 0.41, *p* < 0.001; *V*_slope_= 15.87, *SE* = 5.82, *p* = 0.006). Furthermore, there was a significantly negative quadratic change (*M* _q_ = −0.10, *SE* = 0.04, *p* = 0.02; *V*_q_= −0.21, *SE* = 0.10, *p* = 0.03), implying the increase in IGD has slowed and leveled off over time. The association between slope, quadratic change, and intercept is significant (*r* = −48.82, *p* < 0.001; *r* = 4.70 *p* < 0.001), suggesting that adolescents with a higher baseline level of IGD tend to exhibit smaller growth and higher quadratic change in their IGD level from sixth to ninth grade. The correlation between slope and quadratic change is not significant (*r* =−0.49, *p* = 0.45).

### 3.3. Relationship between Gender and Adolescents’ Developmental Trajectories

**Figure 2:**
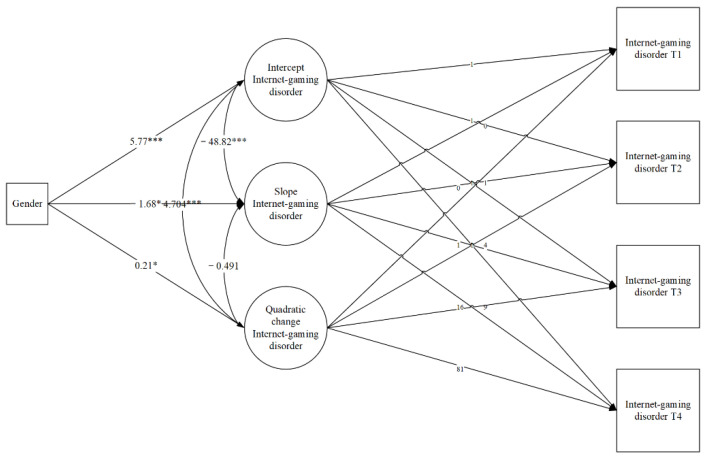
Quadratic LGMs with a time-invariant covariate. * *p* < 0.05, *** *p* < 0.001.

This study examined the association between gender and the baseline level (intercept), change over time (slope), and quadratic change in IGD (Figure 2). The model fit is good: χ^2^ = 1.254, *df* = 2, *p* = 0.53, *CFI* = 1.000, *TLI* = 1.022, *SRMR* = 0.017. Gender could significantly predict the baseline level (*β* = 5.77, *p* < 0.001), IGD increase (*β* = −1.68, *p* = 0.039), and quadratic change (*β* = 0.21, *p* = 0.014).

### 3.4. Time-Specific Effect of Depression Status on Adolescence’s Internet Gaming Disorder

**Figure 3:**
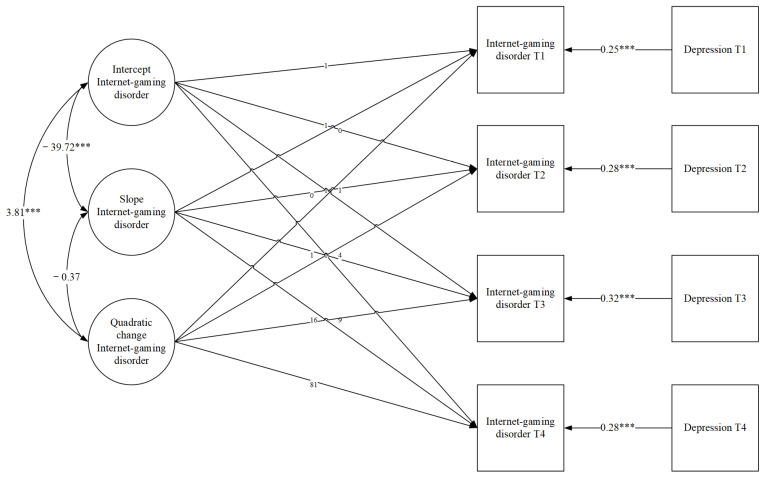
Quadratic LGMs with time-varying covariates. *** *p* < 0.001.

The model of adolescents’ IGD with concurrent depression at grade 6–9 as a time-varying covariate (Figure 3) fits the data well: *χ^2^* = 3.79, *df* = 13, *p* = 0.99, *CFI* = 1.000, *TLI* = 1.118, *SRMR* = 0.046. Depression was significantly associated with IGD at grade 6–9 (*β* = 0.25 *p* < 0.001; *β* = 0.28 *p* < 0.001; *β* = 0.32, *p* < 0.001; *β* = 0.28, *p* < 0.001), which means adolescents with lower depression reported lower levels of IGD. When controlling for variance in depression, the linear change and quadratic change were no longer significant (*M*_s_ = 0.89, *p* = 0.24; *M*_q =_ −0.051, *p* = 0.523).

### 3.5. Trajectories of Depression and Internet Gaming Disorder in Early Adolescence

**Figure 4:**
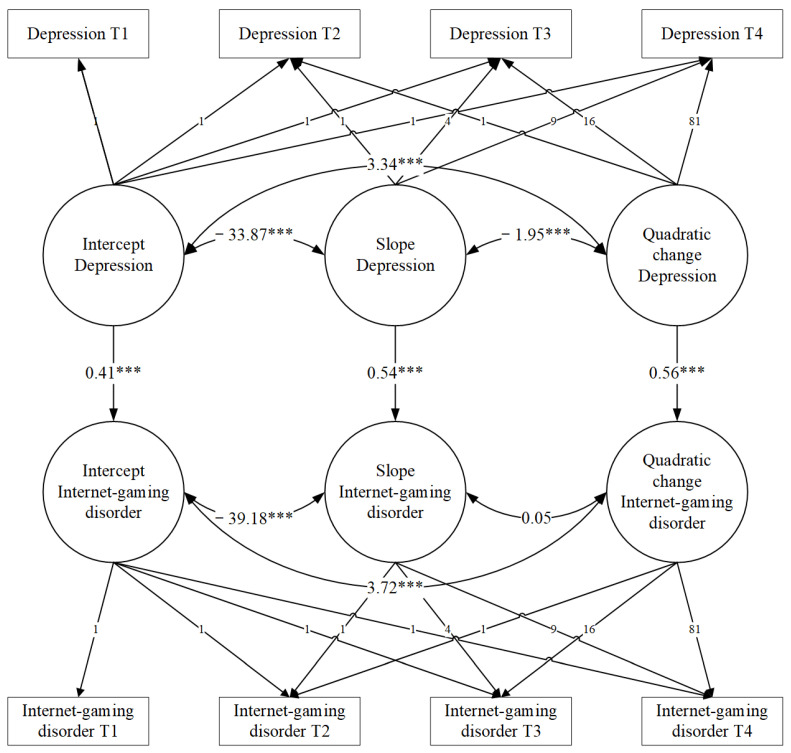
Unconditional LGM with multiple growth process of depression and Internet gaming disorder. *** *p* < 0.001.

To examine whether depression development could explain IGD change, we estimated an unconditional LGM with the multiple growth process model (Figure 4). This model contains repeated measurements of depression and IGD and assumes that the trajectories of adolescents’ depression and IGD are mutually correlative.

Firstly, the quadratic model (*χ^2^* = 2.37, *df* = 1, *p* = 0.12, *CFI* = 0.901, *SRMR* = 0.049), which depicted trajectories of adolescents’ depression, fitted the data better than the linear model (*χ^2^* = 39.73, *df* = 5, *p* < 0.001, *CFI* = 0.65, *SRMR* = 0.182). Furthermore, according to Wang (2011), the covariance between the latent intercept development factors of the two trajectories (depression and IGD) provided evidence of a coexistence relationship of the two variables at the beginning of observation [36,37]. The similar correspondence is also present in slope and quadratic change. The results of this model could reveal that the causality between depression and IGD, which is supported by the theoretical conditions discussed in the introduction.

The unconditional LGM with multiple growth processes fits the data well: χ^2^ = 8.679, *df* = 15, *p* = 0.89, *CFI* = 0.98, *TLI* = 0.92, *SRMR* = 0.074. Aligned with our expectation, the depression level at sixth grade positively predicted the intercept of IGD (*β* = 0.41, *p* < 0.001), suggesting that highly depressed adolescents tended to exhibit IGD. The slope of depression could be a predictive index for the slope of IGD (*β* = 0.54, *p* < 0.001), suggesting that early adolescents who exhibited a higher growth rate in depression tended to exhibit a higher growth rate in IGD. Finally, the quadratic change in depression significantly predicted the quadratic change in IGD (*β* = 0.56, *p* < 0.001); those with a faster change rate of depression reported a faster change rate of IGD.

## 4. Discussion

### 4.1. The Trajectory of Internet Gaming Disorder in Early Adolescents

In this study, the LGM of the development trajectory of IGD in early adolescents is the growth model of nonlinear latent variables. The intercept of the unconditional nonlinear LGM (starting level of sixth grade) of the IGD is 20.76, significantly greater than 0, indicating that the IGD had developed significantly when teenagers were first measured in the sixth grade (*M_age_* = 11.63 ± 0.64). This is consistent with the findings that adolescents around 14 years of age meet the diagnostic criteria for or are at risk of IGD.

The slope of the unconditional nonlinear LGM of IGD is 1.50, and statistically significant, indicating that the level of the IGD in the early stages of adolescence is on the rise. Using a cross-lag board design, Wartberg et al. (2019) showed that the IGT at T1 significantly positively predicted IGD at T2, that is, IGD is on the rise [22].

The slope of the unconditional nonlinear LGM of IGD is −0.10, and statistically significant, indicating that although IGD in early adolescents is on the rise, and its growth rate is gradually slowing, which has not been revealed in previous studies.

In addition, the results of data analysis reveal that the initial state of IGD in early teens (sixth grade) is significantly negatively correlated with their development trend, and their quadratic change is significantly positive, indicating that the increase in young people with a low starting level of IGD is greater than that of individuals with a high starting level; with time, its growth rate is slower than those with a high starting level of IGD.

### 4.2. Gender Prediction of Internet Gaming Disorders

As a time-invariant covariate, gender can significantly positively predict the starting state and rate of change of Internet gaming disorder in early adolescents, which means that, in early adolescence, boys start at a higher and faster rate than girls. This is consistent with the higher incidence of IGD in men than in women in studies on adults [27].

Gender can significantly negatively predict the rise in IGDs in early teens, which means that girls are more likely to experience a larger increase in IGDs in early teens than boys. This is consistent with the results of another study (Phan et al., 2020) showing that, before and after the age of 15, symptoms of IGD were prevalent in men at 21% and 19% and in women at 6% and 7%, respectively [38], although the differences were not statistically significant, but the trends were consistent with those of the current study.

Gender was a significant positive predicter of quadratic change, meaning that, in early adolescence, the rate of development of IGD in girls is slower than in boys over time. This is a new finding compared with previous research.

### 4.3. Depression Predicts Internet Gaming Disorder

In early adolescence, as a time-varying covariate, depression levels at each measuring point in time can positively and significantly predict the level of IGD at each point in time. This is consistent with previous studies that find depression is significantly associated with IGD [10,16].

To further explore the effect of depression development trajectory on the development trajectory of IGD in early adolescence, an unconditional LGM with multiple growth processes was constructed. The results support the theoretical hypothesis of the I-PACE model that depression can be incorporated as a psychological factor into the P component of the model (a susceptibility variable representing core personal characteristics) to predict specific network disorder behavior [4].

In the early stage of adolescence, the intercept, slope, and curve slope of depression development trajectory can predict the intercept, slope, and curve slope of the IGD, respectively. That is, individuals with a high initial level of depression usually have a high initial level of IGD, individuals with a large range of depression display a large range of IGD, and those with a rapid rate of depression change show similar change in their IGD. This further proves the causal relationship between depression and IGD.

### 4.4. Research Significance

With the development of the Internet, gamers spend an average of more than six hours a week on games, and studies have shown that certain types of online games have a high potential for addiction [39]. A two-year longitudinal study of primary and secondary school students revealed that the prevalence of pathological IGDs was approximately 9% among such students [40].

This study tracked the development of IGD in early adolescents and described it in more detail and accurately through the LGM, thus providing an empirical basis for the prevention and intervention of IGD in early adolescents. Firstly, the trajectory of Internet gaming disorder in early adolescents can show that IGD is quite high in grade 6, which means that special attention should be paid to IGD in adolescents starting in sixth grade, such as limiting the time spent on smartphones and other electronic devices. Secondly, gender prediction of IGD points out that boys and girls develop IGD differently, which means that gender differences should be paid attention to when preventing and intervening with IGD. For example, group psychological counseling can be divided into boys and girls groups. Finally, this study suggests that depression predicts IGD. Therefore, in the process of preventing IGD, attention should be paid to some measures to reduce adolescent depression, such as more physical education classes, getting in touch with nature, and so on, so as to reduce IGD.

At the same time, although the relationship between depression and IGD is clear [17,18], the specific predictive relationship between the two variables remains vague [14,26,27].

Based on the I-PACE model [4], LGM with multiple growth process model analysis is used to provide a longitudinal study of the relationship between IGD and depression levels, further clarifying that depression levels are a predictor of IGD. It also proves the predictive effect of gender as a time-invariant covariate on IGD [26,27,40,41].

### 4.5. Limitations and Prospects

First, the samples in this study came from a general group of junior high school students, not from the clinical environment (individuals diagnosed with IGD), so the results may not be applicable to clinical samples [1]. Future studies can further prove the conclusions of the current study by examining clinical samples.

Secondly, this study lacks the support of the physiological field, and by collecting subjective evaluation data and analyzing individuals, this study lacks the support of objective physiological evidence. Studies have shown that IGD as an addictive behavior can exhibit functional magnetic resonance imaging (fMRI) brain changes [41].

Finally, this study does not fully confirm the I-PACE model [4] as IGD still has many predictors.

## 5. Conclusions

Some new findings in this study show that, in the early stages of adolescence, the development trajectory of individual IGD is a nonlinear LGM, the IGD is clearly evident when teenagers are first measured (sixth grade), and IGD is on the rise, but its growth rate is gradually slowing.

Gender can significantly predict the development trajectory of individual IGD; in early teens, boys’ starting level is higher, the change rate is faster than in girls, and the increase in girls is initially more pronounced.

Early adolescents with a high initial level of depression usually have a high initial level of IGD, and individuals with large changes in depression have a large change in their IGD. Those with a rapid rate of increase in depression have a high rate of change in IGD. Depression is an important predictor of IGD.

## Figures and Tables

**Table 1:** Mean levels and bivariate correlations among main study variables ^1^.

Variables (*N* = 316)	*M* (*SD*)	1	2	3	4	5	6	7	8	9
1. Gender		1								
2. Depression (sixth grade)	15.75 (10.29)	0.023	1							
3. Depression (seventh grade)	14.42 (10.73)	−0.63	0.605 ***	1						
4. Depression (eighth grade)	18.63 (12.41)	−0.021	0.027	0.029	1					
5. Depression (ninth grade)	17.98 (12.59)	−0.010	0.426 ***	0.407 ***	0.070	1				
6. Internet gaming disorder (sixth grade)	20.51 (10.88)	0.018	0.271 ***	0.208 **	−0.079	0.116	1			
7. Internet gaming disorder (seventh grade)	21.50 (10.99)	−0.052	0.215 **	0.351 ***	−0.083	0.150 *	0.519 ***	1		
8. Internet gaming disorder (eighth grade)	25.52 (13.11)	0.129 *	−0.016	−0.073	0.278 ***	0.060	−0.064	0.011	1	
9. Internet gaming disorder (ninth grade)	24.87 (13.77)	−0.072	0.174 *	0.210 **	−0.108	0.273 **	0.207 **	0.497 ***	0.036	1

^1^ Grade 6 to 9 are a form of junior high school in China and the age is around 11 to 14 years old. * *p* < 0.05, ** *p* < 0.01, *** *p* < 0.001.

## Data Availability

The data presented in this study are available on request from the corresponding author. The datasets for this manuscript are not publicly available because the datasets are used only for the team of this article by the permission of the subjects. Requests to access the datasets should be directed to the corresponding author Peng Wang.
